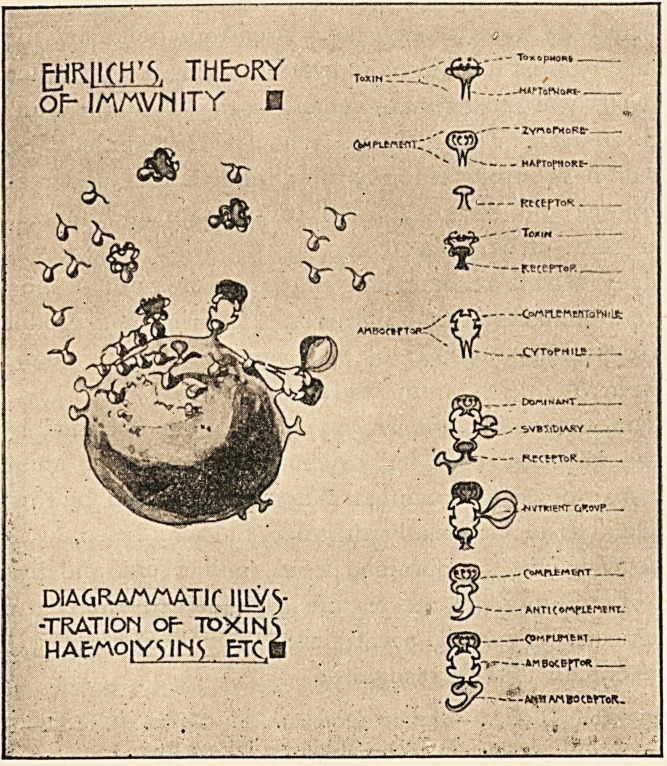# The Antibodies in Disease
1Read at a Meeting of the South-Western Branch of the British Medical Association at Exeter, April 1st, 1903.


**Published:** 1903-09

**Authors:** F. Bushnell

**Affiliations:** Hon. Pathologist, S. Devon and E. Cornwall Hospital, Plymouth; Lecturer on Bacteriology, Plymouth Education Authority.


### THE ANTIBODIES IN DISEASE.[1]

BY

F. BUSHNELL, M.D.Lond.,

*Hon. Pathologist, S. Devon and E. Cornwall Hospital, Plymouth; Lecturer on Bacteriology, Plymouth Education Authority.*

---

ALMOST every current number of the medical journals contain a reference to the subject of my paper, and perhaps illustrates the difficulty that exists in dealing with the antibodies and immunity in the space of twenty minutes, which custom places as our time limit.

From an etymological standpoint I owe you an apology for the use of the weird word "antibody"; but at the same time I know of no other which so clearly expresses the characteristics of these substances; *e.g.*, "antidote" implies something given or administered, and is unsuitable therefore.

The nomenclature is undoubtedly confusing, and I must ask your indulgence for the multiplicity of names given to the same bodies, which I will limit as far as possible.

As the subject is of growing importance to the practice of medicine and the treatment and the understanding of disease processes, it is of interest, I think, to us all. Its origin in the work of Jenner, Hunter, and Pasteur is now a part of medical history. Twelve years ago immunity was explained in three ways. Metchnikoff, a Russian biologist, working in Paris, maintained that leucocytes ingested and digested virulent bacteria in the successful resistance to an invasion of the animal tissues.

Nuttall and Buchner, in 1888, discovered the bactericidal action of the blood, and the two theories were known as the cellular and humoral.

Others, as Hankin, believed in a cellulo-humoral theory, or combination of the two, and all evenly-balanced minds will accept at the present day this explanation. Indeed, Metchnikoff

[1] Read at a Meeting of the South-Western Branch of the British Medical Association at Exeter, April 1st, 1903.

has now ingeniously adapted his cellular theory to the humoral one (so lucidly set forth of late by Ehrlich); and Kanthack some years ago showed that protective substances, or alexins, existed in the eosinophil granules of leucocytes. In 1891 Behring discovered the antitoxins of diphtheria. He found that the *serum* of animals which had been "hyper-vaccinated," or immunised against diphtheria by previous injections ot diphtheria bacilli, or their toxins, had the power of protecting other animals against diphtheria, when injected into them.

Pfeiffer discovered the lysins or dissolvers of bacteria in 1894. He injected cholera spirilla into the peritoneal cavity of a guinea-pig which had been vaccinated or immunised against cholera, and withdrew a little of the culture at short intervals. The organisms became granular, then rounded, and finally disappeared entirely, and the animal survived. This experiment, known as "Pfeiffer's phenomenon," is a process of digestion, and explains bacteriolysis, and also the successful resistance to the invasion of the bacteria of the disease in question afforded to animals or persons treated with immune sera.

This led to the investigation of the action of sera of animals, immunised against foreign *cells* by their injection, upon these cells; *e.g.*, rabbit's serum immunised against fowl's corpuscles dissolves fowl's corpuscles; and analagous results were obtained by Bordet, Metchnikoff, Morgenrorth, and Ehrlich who demonstrated the nature of this process, or hæmolysis.

Metchnikoff amplified Pfeiffer's phenomenon by obtaining it in vitro; mixing immune serum, peritoneal exudate and bacteria, and obtaining bacteriolysis. Bordet showed that peritoneal exudate could be replaced by normal serum and similar results obtained, and also that a little fresh serum would renew the activity of the immune serum, or "reactivate" it, if it were at all lessened.

Thus was shadowed out the co-operation of two substances in the process of bacteriolysis. A further step forward was then made. Bordet proved that heating the immune serum to 65° C. did not affect its bacteriolytic powers, though it still needed the addition of normal serum. Heating the latter to 55° C., however, prevented solution of bacteria. It was concluded, therefore, that the immune serum contained a stable substance of specific "anti-"nature, and that normal serum contained a labile substance acting as a complement.

Metchnikoff states that the large mononuclear cells or macrophages, and the polymorphonuclear or microphages contain this complement or cytase. Histology shows us that the former cells are associated with resistance to the bacteria of chronic diseases, such as tubercle and leprosy, and to malarial and other parasites, and also to animal cells, as red blood cells; while the latter are of course associated with acute bacterial infections. He considers that these leucocytes secrete a cytase or dissolving ferment, and in an immune animal a special body (fixateur) sensitises the foreign cell to the action of the cytase or complement.

We have now arrived at the stage when two great groups of antagonistic bodies had been separated. (1) *The antitoxins*, which combine with and neutralise toxins; (2) *The lysins*, which act by the co-operation of two bodies, and can digest or dissolve cells in the presence of suitable substances (alexins).

Add to these two great groups two lesser ones, the agglutinins and the precipitins or coagulins, and the list is fairly complete.

The *agglutinins* have the power of clumping those bacteria or cells, the previous injection of which led to their formation; and the *precipitins* similarly form insoluble compounds with the proteids which led to their production.

The *agglutinins* were demonstrated in 1889, when Charrin and Roger observed that b. pyocyaneus, grown in the serum of an immunised animal, formed a deposit at the foot of the tube. Grüber and Durham, in 1896, when investigating Pfeiffer's reaction, observed a similar phenomenon, which Widal reversed and applied to the recognition of disease (typhoid).

The reaction of agglutinins is also seen when one animal's cells are injected into another; *e.g.*, the serum of a rabbit, treated with fowl's corpuscles, clumps fowl's corpuscles (in the absence of alexins).

Precipitins were observed in 1899, when Tchistovitch immunised an animal against eel's serum, which is poisonous, and then found that this animal's serum could give a precipitin with eel's serum. Similarly, goat's serum immunised against horse's serum will give a precipitin with horse's serum.

These results harmonise with the biological test used by Nuttall, Wassermann, and Walter Myers to determine the source of a blood. The blood reacts always with the specific precipitins.

A brief account of the *toxins* is perhaps requisite before dealing with antitoxins. Brieger first isolated from cultures of bacteria certain nitrogen containing bodies, which he called ptomaines; certain similar bodies occurring from metabolic processes in the body being known as leucomaines. But it was remarked that ptomaines never reproduced the symptoms of disease associated with the bacteria from which they were obtained (with the exception perhaps of tetanus).

Roux and Yersin marked a new step by obtaining from a culture of tetanus, filtered through unglazed porcelain, a fluid or toxin which reproduced the symptoms of tetanus. This fluid was sensitive to light and heat; was precipitated by alcohol, and was named toxalbumin. Subsequently, toxalbumoses were obtained from B. tetanus, typhosus, cholera-vibrio and staphylococcus pyogenes aureus.

Toxins are known as intra- and extra-cellular. Thus dead t. b. are very toxic; the new tuberculin T R is their expressed juice. Dead cholera and typhoid bacilli are also most toxic. If cultures of t. b. or b. mallei are boiled, a fever-producing substance may be obtained, such as "old" tuberculin, or mallein.

Professor Sidney Martin was the first to prove that diphtheria bacilli, grown in albumin, produced albumoses, peptones, organic acids and salts with various poisonous actions. He also found toxalbumoses in anthrax, ulcerative endocarditis and tetanus. The chemical nature of toxins is still uncertain, and varies markedly. They can be "salted out" fractionally from the various proteids of immune serum. The virulence of an infinitesimal dose is sometimes extraordinary, and reminds one of a ferment. Quite similar vegetable and animal poisons exist, viz., ricin, abrin, rabin, and venin. All toxins possess two characteristics, viz., they produce poisonous symptoms and combine with antitoxin.

Professor W. H. Welch, in his Huxley lecture,[1] makes the interesting suggestion that bacteria produce toxins as an antibody to the animal cell, just as the animal cell manufactures antibodies to the bacterium; on the ground that action and reaction are equal. By far the most valuable observations as to the constitution of toxins, and explanation of their combination with antitoxins, is that afforded by Ehrlich's side-chain theory.

During the difficult process of attempting to standardise diphtheria toxin, certain anomalous results were obtained. I venture to remind you first that the U of T has been defined as the smallest amount of T which kills a guinea-pig of 250 gms. weight in four days; it is also known as the M L D. Also that the U of A has been arbitrarily fixed as the amount which exactly neutralises 100 U of T. A "normal" unit of A is one in which 1 c.c. contains 1 unit.

Ehrlich took a U of A serum (able to neutralise 100 M L D according to our definition) and added successive quantities of toxin to it. This mixture (in which a U of A exactly neutralised a certain amount of T, when injected into a guinea pig) he called the L O dose. On adding a lethal dose of T to this, you might well expect that the guinea pig would die in four days, but it does not do so—though œdema and paralysis may occur. The exact amount of T necessary to produce a lethal effect in four days he called the L + dose, (sometimes = 28 M L D.

Ehrlich found then (*a*) That of various T a quantity containing less than 100 lethal doses often neutralised the U of A, though on the other hand it might contain even more, yet neutralise one unit only, *e.g.*, 20 or 130 M L doses respectively; (*b*) That the excess of toxin required to be added to the neutral mixture to produce a lethal effect was more than one lethal dose. (*c*) That if you keep a sample of T, its lethal dose might be reduced, say, to one-third, whereas its L O or neutralising dose remained unaltered (this though the A had undergone no change). The explanation he gives is that there exists a modification of toxin called *toxoid*,

[1] *Johns Hopkins Hosp. Bull.*, 1902, xiii. 287.

which is almost without poisonous effect, but which can unite chemically with A. The *toxoids* may be grouped into proto-, syn-, and epi- toxoids, according as their affinity for A is more than equal to or less than T. When the affinity is less than T, the epitoxoid will be set free, and until all of the *toxoids* are set free no *toxin* is said to be set free. You have, therefore, a mixture of A and T, containing possibly toxoids, capable of neutralising one another exactly; on adding a dose of toxin (perhaps containing several lethal doses) the epitoxoids are turned out from their union with antitoxin by the bodies possessing greater affinity for A, thereby enabling more T to be anchored and neutralised, and largely increasing the difference between L O and L + doses. This is the way it presents itself to my mind, and is the best mental picture I can construct.

Toxins may become toxoids by various agencies, and toxoids are capable of producing immunity, and are not very poisonous. It has been shown that the union of toxin and antitoxin occurs in multiple proportions or fixed ratios, and more rapidly in warm solutions; that diphtheria toxin passes through a gelatine film, while its antitoxin cannot do so; yet the filtrate of a mixture is not toxic, or but little so; and, finally, that tetanus toxin and emulsion of the central nervous system is practically inactive.

All these facts prove that the union of T and A and T and body cell is chemical, and Ehrlich puts forward a side chain theory, borrowed from organic chemistry, to account for their combination. The ultimate molecule of T has two affinities or side chains, one known as the haptophorous and the other as the toxophorous. On the latter the poisonous action of the toxin depends. When circulating in the blood the T finds a side chain, which fits its haptophore group and unites with it. If it is fixed to a body cell its poisonous group now comes into play, and possibly, like a ferment, begins to digest the very cell, which was seeking food perhaps! When, however, the T meets, as in A, with a suitable side chain, it is neutralised thereby.

On this reasoning both the poisonous actions of the T and the neutralising action of A depend upon the use of a probable physiological function by pathological substances. The function in question is that concerned in the assimilation of proteid food, according to Ehrlich; and without this potentiality poisoning by toxins, etc., could *not* occur. Nevertheless, out of evil may come good, for the T may stimulate the body cells to such an extent that they throw off these side chains, which pass into the blood as A. The A have one side chain, but the cytolysins have two, as also the agglutinins. On these grounds we should expect, as antibodies exist in the body preformed, that they might be found adrift sometimes in normal blood, and this is the case.

I quote to you two examples from the last Huxley lecture of Professor W. H. Welch[1] of the application of this interesting theory. The blood in infancy and adult life varies with health, nutrition, alcohol, inanition, pain, and disease as to its antibody constituents, especially its alexins. The infant comes into the world with a less amount of protective antibodies in the blood than the healthy adult, but they are supplied by the maternal milk. This explains at the same time, the greater freedom of the suckling from infectious disease, and, to my mind, also the high infant mortality among bottle or hand-fed infants. Again, in chronic diseases of the heart, blood-vessels, and kidneys, the frequency of terminal bacterial infections is explained by the known diminution of antibodies.

It appears to me not to be impossible that this theory might be extended to explain the immunity that exists in habitual opium eaters to the drug, and to morphia, to arsenic, to alcohol, and even to the ingestion of impurities in air, food, and water by some individuals with apparent impunity. There is no doubt of the affinity of these poisons for certain tissue cells, and in proportion to their absorption, such combination would presumably occur; but their toxic powers are obviously greatly lessened in the cases I have mentioned, without a diminution in their poisonous chemical properties.

How does this occur? Are antibodies produced and circulating in the blood of those who habitually consume with impunity enormous doses of opium, arsenic, or alcohol?

[1] *Loc. cit.*

Is it unreasonable to expect that the haptophore groups of such metallic, alkaloidal, or organic substances might over-stimulate the tissue cells to produce anti-metallic or anti-alkaloidal bodies? I submit that experiments on this point might furnish useful knowledge one way or another, especially to chemical pathologists.

For my own part, I am so convinced also of the important part played by the mind and nervous system on the body and disease, in predisposing to attack, that I should expect a complete theory to explain its relations.

I may recall to you that immunity may be natural or acquired (artificial), and that artificial immunity may be active or passive. Active immunity against diseases may be brought about by the direct injection of *organisms or their toxins*. Passive immunity by the injection of the *serum* of a *highly* immunised animal into another, its effect being antitoxic or antibacterial, agglutinating or precipitating.

Among the important practical applications of the principles of active immunity are:—

(*a*) The protective inoculation of sheep and oxen against anthrax (Pasteur).

(*b*) Vaccination against small-pox (Jenner).

(*c*) Anti-cholera inoculation (Haffkin).

(*d*) Anti-plague inoculation (Haffkin).

(*e*) Anti-typhoid inoculation (Wright and Semple).

(*f*) Inoculation against hydrophobia (Pasteur).

Of these, the value of *b* and *f* may be said to be fully established.

Among the important applications of *passive immunity* are the use of *antitoxic* sera of diphtheria, tetanus, snake-poison, ricin, abrin, and, lately, cholera; while the antibacterial or cytolytic sera have been used in typhoid, cholera, bubonic plague, b. coli communis, pneumococcus, streptococcus and other infections.

To reiterate, you know that the serum of an animal *A* treated by repeated injections of toxin may protect an animal *B* against a certain amount of this toxin, when injected with, shortly before, or after it. The serum of A neutralises the toxin, and is called antitoxic.

The *preparation* of such sera is familiar to most of us from business advertisements. They are, briefly, the preparation of a powerful toxin, its standardisation, the production of antitoxin by injection of the toxin into a suitable animal, and the standardisation of the antitoxin.

To give a familiar example :—

(*a*) For diphtheria toxin the bacilli are grown in alkaline albuminous mediums in shallow flasks, for tetanus the growth is made in glucose, both in a warm atmosphere. It is then filtered through a Chamberland filter. (*b*) The strength of the toxin is, for practical reasons, estimated by comparing it with A of known strength, rather than by finding the M L D. (*c*) Antitoxin is usually obtained from horses by injecting them with these toxins, at first subcutaneously, and finally into the jugular vein. After three months' treatment, the dose of 300 c.c. of toxin may be borne by a healthy horse of good resistance. (*d*) "A" must then be standardised. As it has no effect on a healthy body, this is not easy. A certain bulk of it must be examined against a toxin of known strength. As toxin does not keep well, you must first standardise the T against an A of known strength. (Ehrlich uses 1700 I U in 1 gm.) When the power of A is strong enough, the animal is bled under aseptic precautions, and serum allowed to separate and mixed with 0.5 per cent. of carbolic acid.

Sidney Martin recommends that 4,000 units of A should be at once administered in diphtheria. Behring has produced a serum containing 3,000 U of A in 5 or 6 c.c. bulk. 20 c.c. is a maximum volume to be injected at one place.

Again, the *antibacterial or cytolytic* sera are obtained by the repeated injections of living virulent bacteria into an animal *A*, which may protect an animal *B* against infection by the same organism. It is not usually antitoxic, but may be so.

The *agglutinins* are obtained from similar sera, and by their power of "clumping" the organism concerned afford a means of diagnosis. Typhoid, paratyphoid, b. coli communis, Malta fever, etc., can thus be recognised.

A.—Antitoxin. U of A.—Unit of Antitoxin. T.—Toxin. U of T.—Unit of Toxin. M L D.—Minimum Lethal Dose.

As to the results obtained by the use of sera, the value of diphtheria antitoxin may be taken as proved: of 7,000 cases collected by Loddo, the mortality was 20 per cent., as compared with a former mortality of 44 per cent. in the same hospitals. Chronic tetanus, plague, streptococcal and pneumococcal antisera are upon their trial.

Vaccination against typhoid is on the whole favourable to the prevention of the disease and mildness of its course.

Of 49,600 persons, 8,600 were vaccinated with a case incidence 2.2 per cent., and a mortality 12 per cent.; of 41,000 unvaccinated, case incidence was 5.7 per cent. and mortality 21 per cent. (Wright).

The cytolysins are, according to many, the most likely bodies to look to in the future for the successful treatment of cancer.

I am indebted to the kindness of Dr. A. S. F. Grünbaum, F.R.C.P., for permission to make use of this diagram.

## Figures and Tables

**Figure f1:**